# A highly divergent sample from a nearly extinct SARS-CoV-2 lineage in a patient with long-term COVID-19

**DOI:** 10.3389/fcimb.2025.1623390

**Published:** 2025-09-19

**Authors:** Elena Nabieva, Galya V. Klink, Andrey B. Komissarov, Stanislav V. Zaitsev, Maria Sergeeva, Artem V. Fadeev, Kseniya Komissarova, Anna Ivanova, Maria Pisareva, Kira Kudrya, Daria Danilenko, Dmitry Lioznov, Ryan Hisner, Federico Gueli, Thomas P. Peacock, Cornelius Roemer, Georgii A. Bazykin

**Affiliations:** ^1^ Russian Academy of Sciences, Moscow, Russia; ^2^ International Laboratory of Statistical and Computational Genomics, HSE University, Moscow, Russia; ^3^ Smorodintsev Research Institute of Influenza, Saint-Petersburg, Russia; ^4^ Kaluga Regional Specialized Centre for Infectious Diseases and AIDS, Kaluga, Russia; ^5^ University of Cape Town, Rondebosch, South Africa; ^6^ Independent Researcher, Como, Italy; ^7^ Department of Infectious Disease, Imperial College London, London, United Kingdom; ^8^ Biozentrum, University of Basel, Basel, Switzerland; ^9^ Swiss Institute of Bioinformatics, Lausanne, Switzerland

**Keywords:** persistent COVID-19, intrahost evolution, immunosuppressed host, gastrointestinal COVID-19, intrapatient evolution of SARS-CoV-2

## Abstract

**Background:**

Although COVID-19 is primarily an acute disease, there are cases of persistent infection, primarily in immunocompromised patients. It is hypothesized that at least some variants of concern (VOCs) have arisen in such persistent cases, with the virus “spilling over” into the general population after accumulating intra-host mutations. Additionally, a growing body of evidence hints at the gastrointestinal (GI) tract as a reservoir of long-term infection, at least in some cases.

**Results:**

We report the genomic analysis of a highly divergent SARS-CoV-2 sample obtained in October 2022 from an HIV+ patient with presumably long-term COVID-19 infection. Phylogenetic analysis indicates that the sample is characterized by a gain of 89 mutations since divergence from its nearest sequenced neighbor, which had been collected in September 2020 and belongs to the B.1.1 lineage, largely extinct by 2022. Of these mutations, 33 were nonsynonymous and occurred in the Spike protein. Of these, 17 are lineage-defining in some VOCs or are at sites where another mutation is lineage-defining in a VOC, and/or have been shown to be involved in antibody evasion, and/or have been detected in other cases of persistent COVID-19; these include some “usual suspects,” such as Spike:L452R, E484Q, K417T, Y453F, and N460K. Molecular clock analysis indicates that mutations in this lineage accumulated at an increased rate compared with the ancestral B.1.1 strain. This increase is driven by the accumulation of non-synonymous mutations, with an average dN/dS value of 2.2, indicating strong positive selection during within-patient evolution. Additionally, the presence of mutations that are rare in the general population samples but common in samples from wastewater suggests that the virus had persisted for at least some time in the GI tract.

**Conclusions:**

Our analysis adds to the growing body of evidence that the evolution of SARS-CoV-2 in chronically infected patients can be a major source of novel epidemiologically important variants and points to the potential role of the GI tract in long-term infection.

## Introduction

Although COVID-19 is typically an acute disease lasting days or weeks, rare cases of persistent SARS-CoV-2 infection are known, and these are usually associated with suppressed host immune systems (Choi et al., 2020; Kemp et al., 2021; Harari et al., 2022; Wilkinson et al., 2022; Stanevich et al., 2023). In such infections, mutation accumulation continues throughout the disease and tends to be faster than in inter-person transmission (Leung et al., 2022; Quaranta et al., 2022; Stanevich et al., 2023). It is hypothesized that this long-term mutational accumulation under weakened immune pressure sometimes allows the virus to evolve the beneficial combinations of mutations with immune evasion potential and that this mechanism could explain the emergence of variants of concern (VOCs) on long phylogenetic branches (Rambaut et al., 2020; Hill et al., 2022; Viana et al., 2022; Neverov et al., 2023). Certain mutations appear repeatedly in different patients with long-term infection; some of them are also lineage-defining in variants of interest (VOIs) or VOCs. The “hotspots” for such mutations are primarily in the Spike gene; Spike:L452R and E484K are prominent examples (Wilkinson et al., 2022). Moreover, the increased substitution rate observed in the Spike protein in some cases of long-term infection mirrors the increased substitution rate in the Spike protein associated with the emergence of the Omicron variant (Ma et al., 2022), lending further evidence in favor of the persistent-infection hypothesis of the origin of Omicron (Du et al., 2022).

While SARS-CoV-2 has primarily been associated with respiratory syndrome, it has been detected in various organs (Van Cleemput et al., 2021; Stein et al., 2022). An early study of intra-host viral evolution in an immunosuppressed patient detected organ-specific mutations, some of which would be lineage-defining in VOCs that emerged after that patient’s death (Van Cleemput et al., 2021). GI symptoms in particular are now considered part of the common COVID-19 symptom set (CDC, 2020). Consistent with this observation is the detection via autopsy of viral RNA in the intestines of some patients (Stein et al., 2022). Intriguingly, wastewater sequencing, which presumably detects virus particles shed through the GI tract, has discovered, at high frequency, certain mutations that are otherwise rare in the general-population sequencing associated with respiratory transmission (Gregory et al., 2022). These mutations may therefore be adaptive specifically to the GI tract. Furthermore, there is evidence that at least in some patients, the virus persists in the GI tract after it is no longer detected oropharyngeally (Natarajan et al., 2022). Together, these findings suggest that the GI tract may act as a sort of reservoir where the virus resides after the acute respiratory phase in at least some long-COVID patients.

Here, we describe the case of an HIV+ patient with a highly divergent SARS-CoV-2 genotype identified in October 2022 that is likely a result of very long-term infection. Interestingly, the patient was initially admitted to the hospital with GI symptoms. Consistent with these symptoms, the numerous mutations private to the patient’s lineage included several that were specific to wastewater sequencing, providing suggestive evidence for viral adaptation to the GI tract. Additionally, the patient’s sample included a number of mutations that are associated with immune escape and/or are lineage-defining in VOCs. Together, these observations lend further support to the hypothesis that VOCs may originate in immunosuppressed patients with long-term infection, and additionally, suggests that the GI tract may sometimes act as a reservoir for such infections.

## Results

### Case description

The female patient was admitted to the hospital in the city of Kaluga, Russia, in September 2022 with symptoms of irritable bowel syndrome, including diarrhea and borborygmus. She had not been vaccinated against COVID-19. Initial screening tests revealed positive results for both SARS-CoV-2, detected by RT-PCR from a nasopharyngeal swab, and HIV, detected by an antigen/antibody HIV assay. The patient (henceforth referred to as “patient K” for “Kaluga”; the identifier is not known to anyone outside the research group and is only used for purposes of this publication) claimed that she had been HIV-positive since 2005 and never received highly active antiretroviral therapy (HAART). The patient also had a dry cough and fatigue but was afebrile. Over the course of her hospital stay, the patient was tested for SARS-CoV-2 by RT-PCR four more times (on days 3, 6, 9, and 13 post-admission), with all results being positive. She has had no recent travel history.

Despite the COVID-19 diagnosis, the patient exhibited no signs of lung injury on X-ray imaging and did not require oxygen support. However, her complete blood count was remarkable for leukopenia (1.6%–2.2%) and a low hemoglobin level (86–108 g/L). On day 14 post-admission, the patient refused any further medical treatment and discharged herself from the hospital.

In April 2023, the patient was admitted to a hospital with diarrhea, myalgia, borborygmus, and progressing fatigue.

### A SARS-CoV-2 sample on an ultra-long branch

We sequenced the COVID-19 sample from patient K obtained on October 2, 2022 (13 days post-admission), and deposited it in Global Initiative on Sharing All-Influenza Data (GISAID) as hCoV-19/Russia/KLU-RII-MH107906S/2022. Bioinformatic analysis of the sequence strongly suggests that it comes from a persistent infection. Specifically, UShER (Turakhia et al., 2021) identified the sample as belonging to the B.1.1 lineage. This lineage has become nearly extinct by late 2022: apart from the variant reported here, it comprised just 0.004% (549 out of 12,648,328) of GISAID sequences with a collection date later than 1 September 2022 (GISAID queried on 18 February 2025), and was only present in 5 of the 76,460 sequences (0.06%) collected at that time in Russia. To verify the phylogenetic placement of the sample in the B.1.1 clade, we considered alternative tools: pangoLEARN (O’Toole et al., 2021) and NextClade (Aksamentov et al., 2021). The latter uses an approach similar to that of UShER, whereas the former employs an entirely different machine-learning method. Both agreed that the most likely placement was B.1.1.

**Figure 1:**
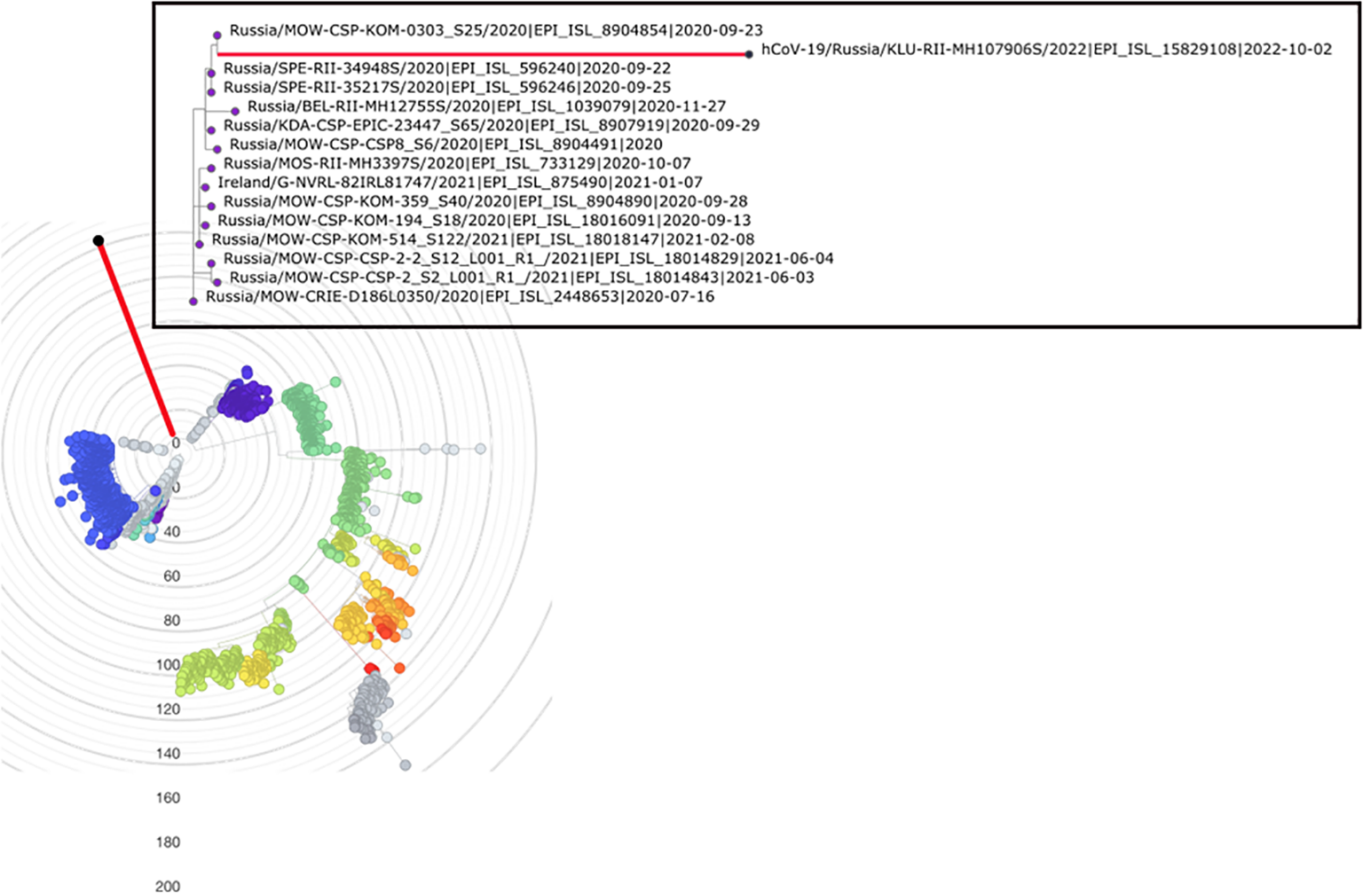
Placement of hCoV-19/Russia/KLU-RII-MH107906S/2022 (red branch) in the phylogenetic tree by UShER (Turakhia et al., 2021), with global tree downsampled (Turakhia et al., 2021) to 2,000 sequences. The inset shows the local phylogenetic vicinity of the patient K sample. Tree visualization by Nextstrain (Hadfield et al., 2018) and Taxonium (Sanderson, 2022).

Despite the robust placement of the sample in the early B.1.1 clade, its lineage is highly divergent, with 89 point mutations since its divergence from the last common ancestor (LCA) with the closest related sample. This closest neighbor, hCoV-19/Russia/MOW-CSP-KOM-0303_S25/2020, was collected in Moscow, Russia, on 23 September 2020 (**Figure 1**). More broadly, out of the 14 phylogenetically adjacent samples, 13 were obtained in Russia between 16 July 2020 and 4 June 2021 (**Figure 1**), confirming that the lineage likely originated from a community infection around the estimated time of the internal node. According to Bayesian estimation with Bayesian Evolutionary Analysis Sampling Trees (BEAST), the median age of the patient K branch was 2.1 years with 90% HPD of 1.8 to 2.3 years. This puts the date of its ancestor to the end of August 2020, consistent with the dating of the nearest outgroup sequences. Together, these observations strongly suggest that this is indeed a case of persistent (up to 2 years or more) SARS-CoV-2. Since we only have a single sample from patient K, it is possible that some mutations unique to its lineage had occurred in previous unsampled hosts. However, mutations accumulated in the general population probably constituted a minority, if any, of the mutations in the patient K lineage, as a lineage circulating for a long time would have been sampled.

### Long-term infection: the most likely explanation for the number of mutations

In the absence of time-course samples from the patient, we establish long-term infection as the most likely explanation for the high number of mutations observed by, first, showing that it could not have resulted from a series of undetected community infections with the normal evolutionary rate, and, second, ruling out the two alternative possibilities that could also explain the “long branch” observation, namely, contamination and recombination.

**Figure 2:**
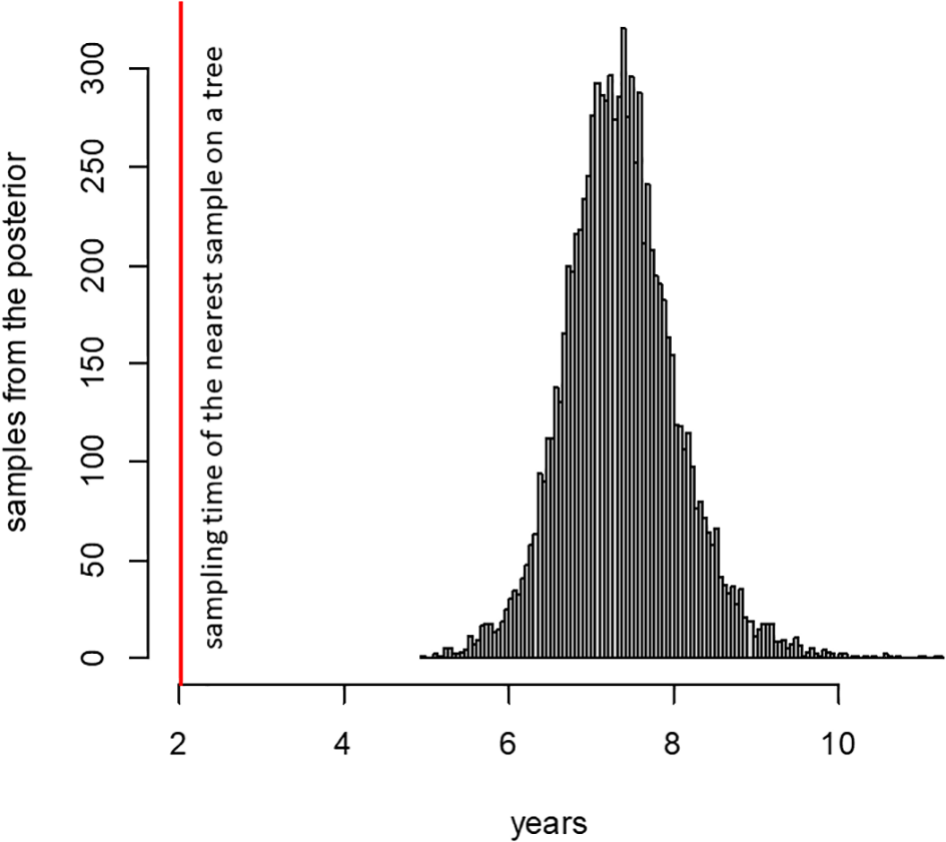
Posterior distribution of the time required for SARS-CoV-2 to accumulate 89 mutations, according to phylodynamic analysis (see text). The red line shows the time period between the sampling dates of the patient K sample and its phylogenetically nearest neighbor (2.02 years).

The 89 mutations observed in the patient K lineage are inconsistent with a series of community infections. To show this, we estimate the rate of evolution of the B.1.1 lineage in Russia outside the patient K lineage using BEAST. Consistent with other estimates (Hill et al., 2022; Markov et al., 2023), this rate is 4.07×10^−4^ (95% HPD 3.32–4.83×10^−4^) substitutions per nucleotide per year. However, if the patient K lineage has also evolved at this rate, this would imply that it had diverged from the last common ancestor with other B.1.1 samples 7.36 years prior to its sampling in 2022 (**Figure 2**). This dating is clearly inconsistent with the phylogenetic placement of the patient K lineage within B.1.1 which originated in 2020 (p < 0.0001), and even with the origin of SARS-CoV-2 itself in 2019 (Pagani et al., 2023). This inconsistency indicates that the bulk of the patient K lineage has been evolving in an accelerated regime, implying that it cannot correspond to a series of community infections. The conjecture that this lineage arose during chronic infection is further supported by its elevated substitution rate (see next section).

We rule out contamination by observing that the sample does not have an excess of variants at intermediate read frequencies. In particular, of the 104 nucleotide point mutations and indels observed in the sample (i.e., having >0.5 of reads supporting them), just a few are at intermediate read frequencies: only 10 have read fractions <0.8, and of these, only 2 have read fractions <0.6.

To see if the observed sequence may be a result of recombination event(s), we ran it through sc2rf (https://github.com/lenaschimmel/sc2rf) to look for evidence of recombination between CoVariants clades (https://covariants.org). As an alternative approach, we used RIPPLES (Turkahia et al., 2021). Neither method detected any recombination events. The “interleaving” of mutations from different VOCs and non-VOC mutations, as well as the presence of non-VOC derived alleles at sites of VOC-defining mutations (**Table 1**), also contradict the hypothesis of the sequence resulting from recombination event(s) involving phylogenetically distant lineages.

**Table 1:** Select Spike mutations in the patient K sample.

Mutation	VOCs with the same mutation or another mutation at the same site in their origin	Immune evasion	Chronic infection	In wastewater	Other notable lineages with the same mutation or another mutation at the same site at their origin
T19A	Delta*, BA.2				
S50L			(Stanevich et al., 2023)		
T95I	BA.1		(Harari et al., 2022; Wilkinson et al., 2022)		
R346K		(Jian et al., 2022; Uraki et al., 2022; Qu et al., 2023)			BA.1.1, *XBB*, *BQ.1.1*
R403K		(Wang R. et al., 2021)			
K417T	Gamma, *Beta*, *BA.1, BA.2*	(Starr et al., 2021; Tada et al., 2021; Wang R. et al., 2021; Wang Z. et al., 2021)			BA.2.38
K444T		(Qu et al., 2023)		(Gregory et al., 2022)	BQ.1
Y449H		(Tada et al., 2022)			
L452R	Delta	(Starr et al., 2021; Wang R. et al., 2021; Takatsuki et al., 2023)	(Wilkinson et al., 2022; Stanevich et al., 2023),		BA.4, BA.5, BQ.1, *BA2.12.1*
Y453F		(Tada et al., 2021)	(Stanevich et al., 2023)		mink cluster V
N460K		(Starr et al., 2021; Qu et al., 2023)		(Gregory et al., 2022)	BA.2.75, BQ.1, XBB
A475S		(Wang R. et al., 2021)			
S477N	BA.1, BA.2	(Wu et al., 2021)			
E484Q	*Beta, Gamma, BA.1,BA.2*	(Starr et al., 2021; Tada et al., 2021; Wang R. et al., 2021; Wang Z. et al., 2021; Tada et al., 2022)	(Harari et al., 2022; Wilkinson et al., 2022)		Kappa
Q498H	*BA.1, BA.2*		(Wilkinson et al., 2022)	(Gregory et al., 2022)	
P621S			(Stanevich et al., 2023)		XAY
N764K	BA.1, BA.2				
Δ141-144	Alpha (Δ144), BA.1 (Δ142-144)	(McCarthy et al., 2021)	(Harari et al., 2022; Wilkinson et al., 2022; Stanevich et al., 2023)		
Δ210		(McCarthy et al., 2021)			

*Lineages where different mutations at the same site were found in the ancestor, or references mentioning different mutations at the same site are italicized.

### Elevated mutation accumulation rate in the patient K sample

**Figure 3:**
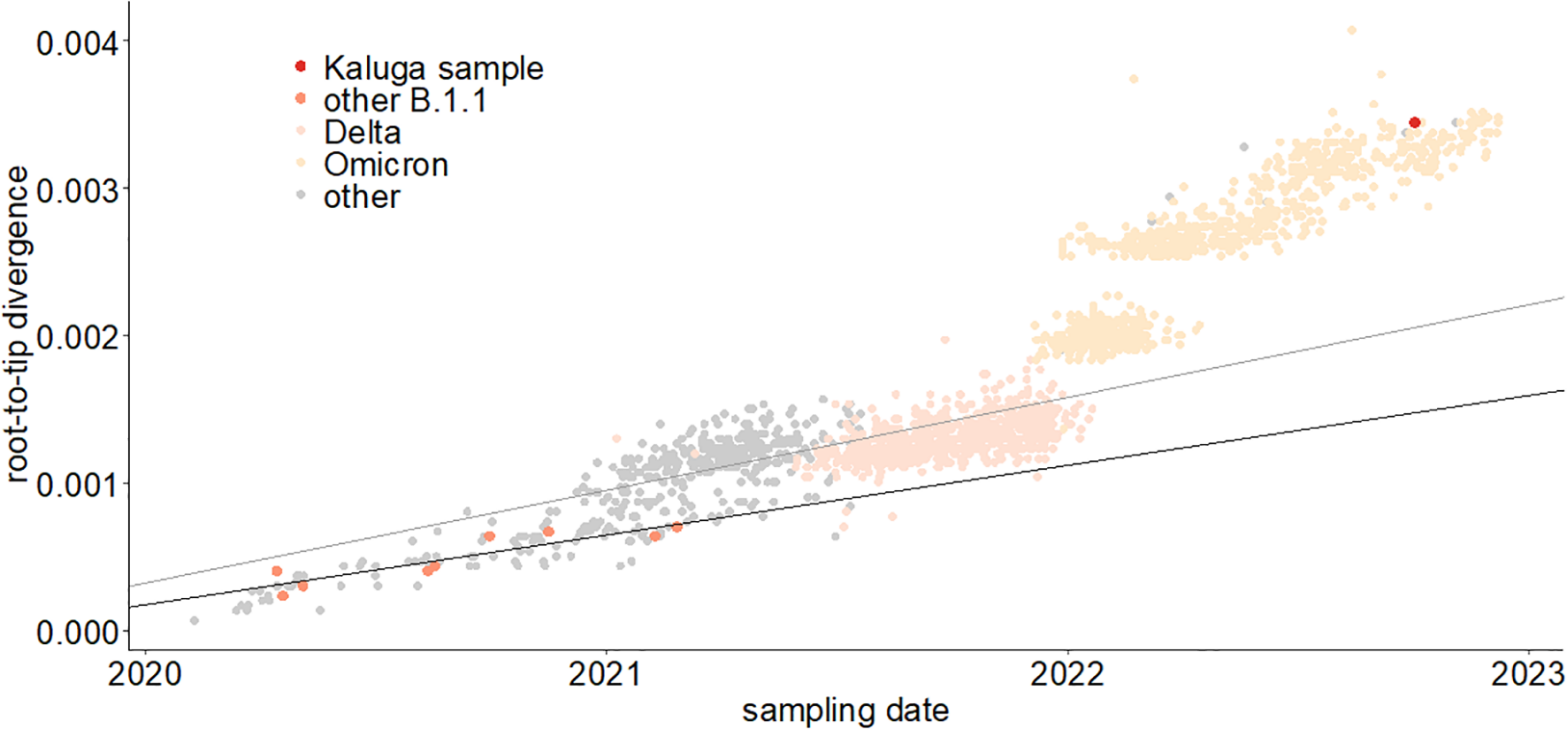
Regression of root-to-tip distance on sampling date. The regression lines are calculated for all non-Omicron, non-patient K samples (thin line, slope = 6.3 × 10^−4^) and just for the B.1.1 samples (thick line, slope = 4.7 × 10^−4^). The patient K sample is high above both regression lines, indicating accelerated evolution at this lineage.

We plotted the number of mutations accumulated since divergence from the Wuhan ancestral sequence for the patient K sample and sequences from the UShER-selected subtree containing it (Turakhia et al., 2021) (**Figure 3**) using TempEst (Rambaut et al., 2016). The rate of mutation accumulation “jumped” dramatically at the beginning of 2022, corresponding to the disproportionately large number of changes at the origin of the Omicron variants (Du et al., 2022; Ma et al., 2022). Remarkably, the patient K sample (red dot) falls on the higher end of the Omicron cloud corresponding to the “BA.2” samples obtained around the same time, even though it belongs to the much more slowly evolving B.1.1 lineage. Thus, the rate of mutation accumulation in the lineage of the patient K sample has been comparable with the rate at the origin of the Omicron clade. Notably, while the rates are similar, the identity of the accumulated mutations is different: the patient K sample shares just a few mutations with BA.2 (see below). The median posterior rate of evolution of patient K lineage was 15×10^−4^ (90% HPD 12–18×10^−4^) substitutions per nucleotide per year, 3.7 times higher than for other B.1.1 samples, which again confirms that this lineage arose during chronic infection.

Single-nucleotide substitutions were non-synonymous (83%), similar to the values observed for samples from immunocompromised patients and from ancestral branches of VOCs (Harari et al., 2022). To evaluate whether the rapid evolution of the patient K sample was caused by positive selection, we applied the Branch-Site Unrestricted Statistical Test for Episodic Diversification (BUSTED) method implemented in HyPhy (Pond et al., 2005). We detected a positive selection on the branch leading to the patient K sample both for the entire concatenated coding sequence (excluding nucleotide positions with overlapping reading frames; dN/dS=2.2 for the branch leading to the patient K sample vs. 0.6 for the rest of the B.1.1 tree, p = 0.04) and for Spike (dN/dS=5.7 vs. 0.7, p = 0.0034). The positive selection (dN/dS > 1) observed for the patient K branch contrasts with the predominantly negative selection (dN/dS < 0.5) observed in the global evolution of SARS-CoV-2 (Rochman et al., 2021). Therefore, the increase in mutation accumulation rate on the patient K branch was likely driven by a positive selection specific to this branch, acting (mainly) on the mutations in the Spike protein.

### Mutations in the Spike protein are typical for long-term infection, immune escape, and VOCs

**Figure 4:**
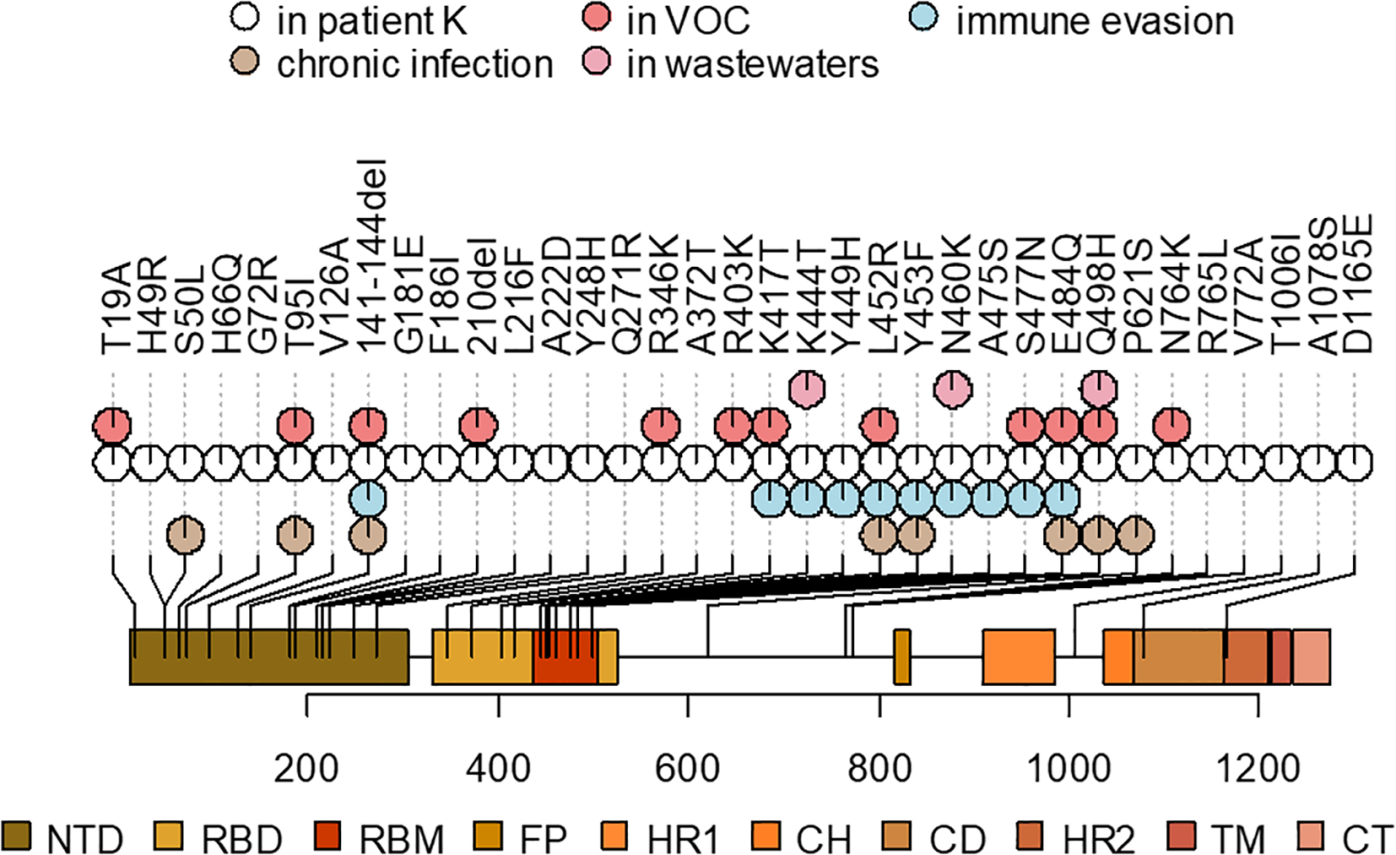
A schematic representation of the Spike mutations in the patient K sample and their overlap with the mutations that were also observed in the ancestors of VOCs, previously described mutations found in chronic infections, associated with immune evasion, or overrepresented in wastewater samples (see **Table 1** for corresponding references). Spike domain coordinates were obtained from ref (Shrivastava et al., 2021).

Of the 89 private point mutations in the sample, 33 occur in the Spike gene, with an additional two in-frame deletions (**Table 1**, **Figure 4**). These include 17 point mutations that have been seen in VOCs or in sites where other mutations have been found in VOCs, and/or in immunocompromised patients, and/or are associated with immune escape. Among these mutations are L452R, E484Q, K417T, N460K, and Q498H. Beyond Spike, the sequence also has several ORF1a mutations that are associated with hypermutated, chronic-infection sequences, most notably T1638I (NSP3:T820I) and K1795Q (NSP3:K977Q) (Wilkinson et al., 2022); the latter has been identified additionally in a number of New York City (NYC) wastewater cryptic lineages, in several of which its fraction was close to 100% (Gregory et al., 2022). These mutations may constitute a response of the virus to selective pressure from the host immune system.

In addition to the point mutations, the sample also contains two deletions in the S gene: Δ141–144 and Δ210. They occur in two of the four recurrent deletion regions (RDRs) in the NTD (RDR2 and RDR3) in the McCarthy et al. (2021) nomenclature and map to distinct antibody epitopes (Wilkinson et al., 2022) and overlap or are adjacent to deletions in VOCs: Alpha (Δ144) and Omicron/BA.1 (Δ211) (Mittal et al., 2022).

The table lists the mutations that are lineage-defining in some of the VOCs or are in sites where another mutation is lineage-defining in a VOC, and/or shown to be involved in antibody evasion, and/or detected in other cases of persistent COVID-19, and/or are characteristic of wastewater samples (see Methods). For the latter three groups, the table cells list the corresponding references.

Other mutations in the S gene are H49R, H66Q, G72R, V126A, G181E, F186I, L216F, A222D, Y248H, Q271R, A372T, R765L, V772A, T1006I, A1078S, and D1165E.

### To the gastrointestinal tract and back again?

The pattern of Spike receptor binding domain (RBD) mutations in the patient K sequence shows a remarkable overlap with the set of mutations that have been repeatedly found in “cryptic lineages” detected in wastewater (Gregory et al., 2022), which are hypothesized to be long-term chronic infections with a GI tropism. Some of them are also found in VOCs, e.g., K417T (Gamma), K444T (BQ.1), and N460K (BA.2.75, BQ.1, XBB), whereas others have been found recurrently in wastewater but are otherwise rare in GISAID. One example is Spike:Q498H, which is found in only 96 sequences in GISAID (accessed on 22 December 2022) but in 7/9 sewersheds studied in (Gregory et al., 2022) (a different substitution at the same site, Q498R, is present in a number of Omicron lineages, and is therefore widespread). This is consistent with the fact that the patient has presented with GI symptoms. Remarkably, around the time of the patient K infection, K444T, N460K, and Q498H had very low frequencies in the GISAID (0.0023%, 0.0026%, and 0.0006%, respectively, as of 15 March 2022 (Gregory et al., 2022)), making their chance of co-occurrence in the same genome negligible, and increasing the likelihood that these mutations constitute a GI adaptation.

Moreover, of particular interest is the double mutation in codon 828 of Spike (CTT->TTG). Conceivably, during the presumed long intra-host evolution, the sequence could go through the intermediate state of TTT (Spike:L828F), which was later followed by the F828L reversion to a different leucine codon. L828F is remarkable in that it comes up repeatedly in cryptic lineages found in wastewater, while being extremely rare in general SARS-CoV-2 sequences (almost exclusively sampled from the respiratory tract) (Gregory et al., 2022). We propose that L828F may represent a specific adaptation to the GI tract during chronic infection. Based on this proposed chain of events and on the overlap with other mutations recurrently found in wastewater samples described above, we suggest that the virus has resided within and adapted to the patient’s GI tract during some period of time in the long course of the infection. Eventually, however, the 828L reversion restored the consensus sequence, presumably better adapted for nasopharyngeal localization, and the virus was ultimately detected in the nasopharynx. Admittedly, this suggested mechanism is highly speculative: the 828F variant by itself was not observed in the patient K sample, the double CTT→TTG mutation could have proceeded through the synonymous (L) CTG rather than the non-synonymous (F) TTT intermediate, and the pathway for the reverse transfer of the GI-adapted variant into nasopharyngeal localization is unclear. Still, this finding adds further, albeit circumstantial, evidence to the idea that a subset of chronic infections may be GI (likely accounting for cryptic wastewater lineages). At present, it is thought that such GI chronic infections likely represent an evolutionary dead end due to scant evidence of transmission of these lineages. However, in this sequence, we find tentative evidence that this virus has re-adapted back to the respiratory tract, giving a potential mechanism for how such a virus could re-evolve to become transmissible by the airborne route.

### Impaired growth in cell culture compared with the B.1 ancestor

**Figure 5:**
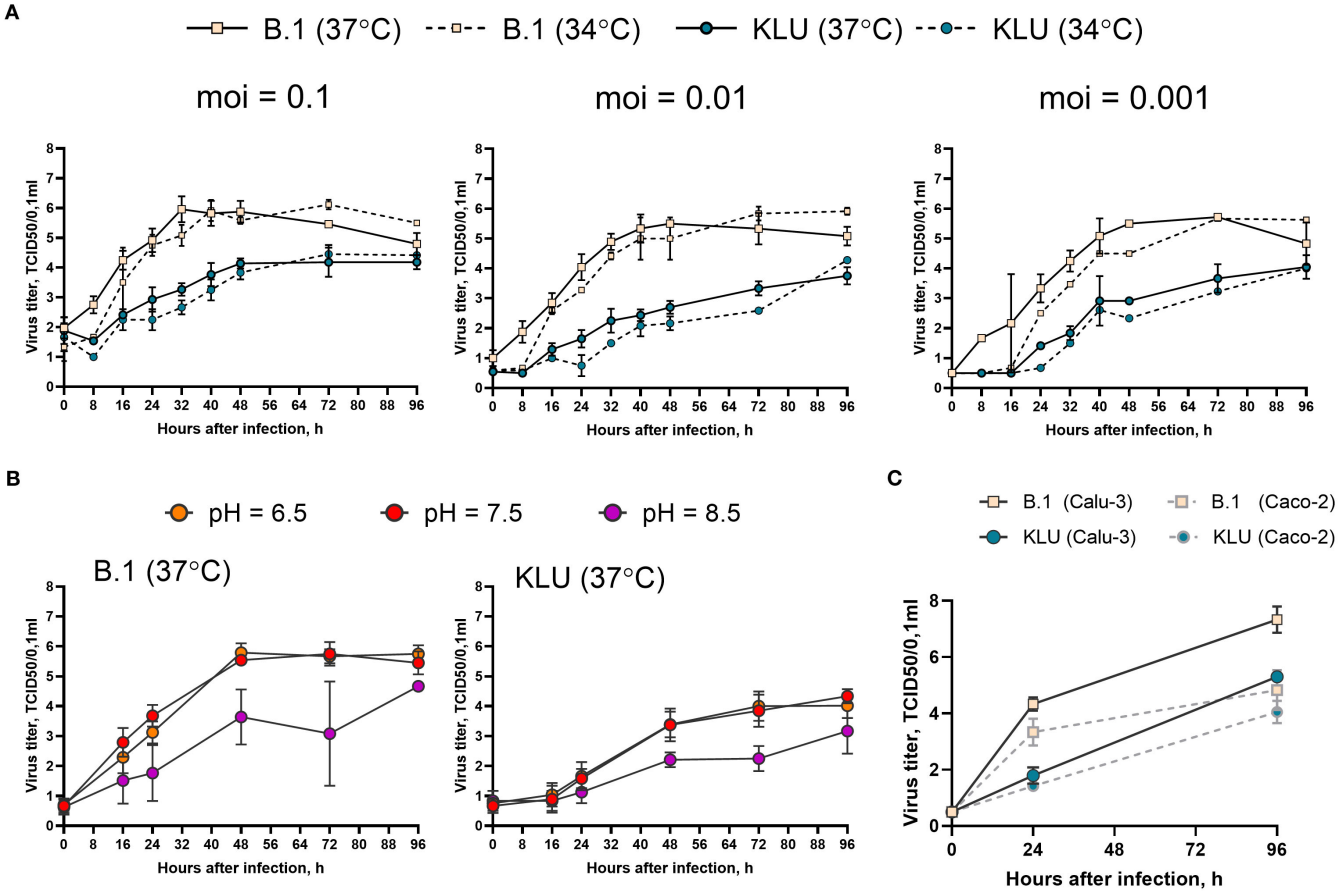
Growth curve of the KLU virus and the B.1 ancestral strain in Caco-2. **(A)** Cells were infected at indicated multiplicity of infection (moi) (TCID50/cell) and incubated at 34°C or 37°C. **(B)** Cells were infected at moi = 0.01 TCID50/cell and incubated at 37°C in the culture medium with indicated pH. **(C)** Calu-3 or Caco-2 cells were infected at moi = 0.001 TCID50/cell and incubated at 37°C. Samples of the culture medium, taken with an 8–24-h interval after inoculation, were titrated for viral infectious activity in the VeroE6/TMPRSS2 cell line.

In an attempt to find particular markers of phenotypic adaptation, we isolated the virus (KLU, sequence deposited in GISAID as EPI_ISL_18647516) from the patient K swab specimen and compared the growth rate of the KLU virus and ancestral B.1 strain in the Caco-2 human intestinal epithelial cell line (ATCC, Cat # HTB-37). First, we checked three different doses (0.1, 0.01, and 0.001 TCID50/cell) and two incubation temperatures (34°C and 37°C). The temperature dependence of the growth rate was the same for both viruses, indicating no loss of adaptation to the upper respiratory site of infection for the KLU virus (**Figure 5A**). Second, we checked three different pH of the culture media: neutral pH = 7.5, acidic pH = 6.5, and alkaline pH = 8.5. The pH dependence of the growth rate was the same for both viruses with no difference between the neutral and low pH and impaired growth at higher pH, contrary to the expectation of adaptation of the KLU virus to the potentially different environment of the gastroenterological tract (**Figure 5B**). For both viruses, no cytopathic effect was observed at any time point. At the same time, we found that the KLU virus growth was overall significantly impaired in comparison with the B.1 strain in the Caco-2 model cell line. The observed difference was similar to that described earlier for the Omicron strains and could be associated with various mechanisms. The first is the previously described greater efficiency of TMPRSS2-dependent cell entry of early lineages of SARS-CoV-2 compared with Omicron (Meng et al., 2022; Willett et al., 2022). We repeated the growth curve experiment in Calu-3 cells, characterized by a different level of TMPRSS2 expression than Caco-2 (Shuai et al., 2020) (**Figure 5C**). Overall, the growth of both viral strains was slightly increased in Calu-3 in comparison with Caco-2 cells; however, nearly the same difference between the strains was obtained in Calu-3, namely, the decreased growth rate of the KLU strain compared with the B.1 virus (**Figure 5C**). The second possible explanation for KLU strain inferiority could be its less effective antagonizing of the interferon response in human cells due to mutations in certain viral proteins (Mautner et al., 2022).

## Discussion

There is growing support for the hypothesis that at least some VOCs, such as Alpha and Omicron, have evolved in patients with chronic infection and escaped into the general population (Rambaut et al., 2020; Hill et al., 2022; Viana et al., 2022). We have described a case of a likely long-term infection in an HIV+ patient. Sampled in 2022, it is most confidently placed in the B.1.1 clade that was largely extinct by that time. In addition to the long branch from the putative root indicative of a long-term infection, remarkable features of the sample include the presence of mutations defining several VOC lineages and/or associated with immune evasion, and an apparently elevated mutation accumulation rate. In fact, the rate is similar to that at the origin of the Omicron clade, suggesting that the evolutionary processes that shaped its evolution may be similar to those that shaped the evolution of the Omicron variant and, conversely, lending further support to the hypothesis that VOCs such as Omicron emerged in the course of chronic infection (Moulana et al., 2022).

It has been shown that many of the lineage-defining mutations in VOCs are associated with antibody escape (providing selective advantage in the largely immune-competent population at the later stages of the pandemic) but that at least some of them reduce RBD affinity for ACE2, an effect that is rescued in BA.1 by the compensatory mutations Q498R and N501Y (Moulana et al., 2022). Neither is observed in our sample, although it contains a different mutation in the 498 codon, namely, Q498H. The rarity of Q498H in GISAID suggests that it may not be responsible for airborne spread, although it has been experimentally shown to increase the ACE2 affinity on a non-N501Y spike background (Mutational effects on ACE2-binding affinity and expression in SARS-CoV-2 variant RBDs). Furthermore, the Y453F mutation greatly increases the ACE2 affinity in both BA.1 and BA.2, as well as Wuhan Hu-1 backgrounds, whereas R403K substantially increases ACE2 affinity on a BA.2 spike background but not a BA.1 or Wuhan Hu-1 spike background (Mutational effects on ACE2-binding affinity and expression in SARS-CoV-2 variant RBDs). Since this sample shares mutations with both BA.1 and BA.2, there is a theoretical possibility of its increased infectivity.

The major limitation of our study is that it is based on only a single sample that was available from this patient. Therefore, the persistence of the virus cannot be verified by repeated positive PCR tests throughout the presumed duration of infection. Instead, the evidence for persistent infection is circumstantial and is based on the likely immunosuppressed (HIV+) status of the patient together with the phylogenetic placement of this sample.

Similarly, the absence of information about the patient’s history (or lack thereof) of COVID-19 infection, as well as the ultimate lack of evidence for community spread of the virus, limit our ability to draw any definitive conclusions about its ability to evade the immune response “in the wild”, beyond the theoretical possibility suggested by the presence of a number of mutations that have been linked to immune evasion (**Table 1**).

The increased fraction of non-synonymous mutations indicates a strong positive selection in the patient K lineage, particularly in the spike protein. The sampled viral genome is characterized by the presence of mutations previously observed in persistently infected individuals. On top of that, an intriguing feature of the patient K sample is the presence of several mutations that have been found almost exclusively in wastewater lineages. This observation suggests the possibility of persistence for some period in the GI tract. Importantly, this suggestion is based on the highly circumstantial evidence and is countered by our finding of lack of pH dependence of the growth rate, which could be expected under GI adaptation. Regardless, the connection between immune-evading long-term infection, GI symptoms, and the risk of emergence of VOCs suggested by the observations in this sample highlight the need for wastewater monitoring.

As of 18 February 2025, the hCoV-19/Russia/KLU-RII-MH107906S/2022 sample remains the sole representative of this lineage in GISAID, although GISAID carries 92,865 additional samples from Russia, including 642 from Kaluga region, with 278 having later sampling dates. Together with the fact that the virus has been sampled from the likely source of long-term evolution (the HIV+ patient), this suggests that this lineage has not led to substantial onward transmission. Still, it indicates that SARS-CoV-2 has not yet run out of opportunities for further adaptation.

## Methods

### Sample collection

Nasopharyngeal swab was collected in virus transport medium. Total RNA was extracted using the AutoPure-96 Nucleic Acid Purification System (Allsheng, China) and NAmagp DNA/RNA extraction kit (Biolabmix, Russia). Extracted RNA was immediately tested for SARS-CoV-2 using Novel Coronavirus (2019-nCoV) Nucleic Acid Diagnostic Kit (Sansure Biotech, China).

The COVID-19 sample from patient K was obtained on 2 October 2022. It had been collected as part of the ongoing surveillance of SARS-CoV-2 variability. The main authors had no access to information that could identify the participant at any stage of the project.

### Sequencing

Whole-genome amplification (WGA) of the SARS-CoV-2 virus genome was performed using the Midnight 1,200-bp amplicon primer set (Freed and Silander, 2021) and BioMaster RT-PCR Premium Kit (Biolabmix, Russia). Library preparation was performed with the Fast PCR-FREE FS DNA Library Prep Set (MGI, China). Finally, sequencing was performed on MGI DNBSEQ-G400 using the FCL SE100 high-throughput sequencing set.

### Bioinformatic analysis

Phylogenetic placement of the K sample was reconstructed using UShER (Turakhia et al., 2021) based on 16,859,038 GISAID, GenBank, COVID-19 Genomics UK Consortium (COG-UK), and China National Center for Bioinformation (CNCB) sequences (accessed on 18 February 2025). Positive selection was tested with the BUSTED (Murrell et al., 2015) method of the HyPhy package (Pond et al., 2005). To allow likelihood inference while preserving the richness of the data, we subsampled the sequences by merging the B.1.1 samples that differed in fewer than five positions with cd-hit (Fu et al., 2012) and then taking a random subset of 500 samples. We then removed samples with premature stop codons, added the patient K sample, and reconstructed a phylogenetic tree with IQTree (Nguyen et al., 2015).

The distribution of evolutionary rates of the Russian B.1.1 clade was estimated using a subset of 100 random B.1.1 samples from Russia (excluding the patient K sample) with BEAST v. 1.10.4 (Suchard et al., 2018) and the following parameters: substitution model GTR+Г4+I, lognormal strict clock prior with mean 0.001, and standard deviation 0.005; 500 million Markov Chain Monte Carlo (MCMC) chain length; the other parameters were default for BEAST, sampling each 50,000th step, for a total of 10,000 samples. The first 10% of steps were discarded before the subsequent analysis. To obtain a distribution of time needed for the accumulation of 89 substitutions, we divided 89 by the rates sampled by BEAST. To obtain the age of the patient K lineage and its evolution rate, we added the patient K sample to this subset and ran the BEAST analysis assuming random local clock with approximate continuous time Markov chain rate reference prior (Ferreira and Suchard, 2008) while keeping the other parameters the same. Again, the first 10% of samples were discarded before the subsequent analysis. The subsequent analysis and visualization were performed in R 4.4.0 (R Core Team, 2013) using the basic functions and the “trackViewer” library (Ou and Zhu, 2019).

### Virus isolation and growth curve

Live virus (KLU) was isolated from the patient K swab samples in VeroE6/TMPRSS2 cells (JCRB, #JCRB1819). Culture was inoculated for 2 h with the swab material diluted 1/10 in Dulbecco's Modified Eagle Medium (DMEM) (Biolot) supplemented with 2% heat-inactivated fetal bovine serum (SC, Biolot, St Petersburg, Russia), 1% anti–anti (Gibco, New York, USA) and then incubated for 4 days until 70% cytopathic effect was reached. The sample was subsequently passaged one time in VeroE6/TMPRSS2 cells to generate the working stock. The sequence of the cultivated virus has been deposited in GISAID (EPI_ISL_18647516).

Growth curve experiments were performed in Caco-2 cells (ATCC, #HTB-37) and Calu-3 cells (ATCC, #HTB-55). KLU virus was compared with the B.1 strain hCoV-19/St_Petersburg-3524V/2020 virus (GISAID EPI_ISL_415710). Cells were grown until 100% monolayer in 12-well plates in Modified Eagle Medium (MEM) culture medium (Gibco) supplemented with 20% FBS (Gibco), 1% GlutaMAX (Gibco), 1% sodium pyruvate (Gibco), and 1% NEAA (Biolot). Monolayer cells were inoculated with a particular viral dose (0.1, 0.01, or 0.001 TCI50/cell) in a 0.5-mL neutral medium with 5% FBS for 1h at 37°C, and then inoculum was removed, and 3-mL medium with 5% FBS was added. The neutral (original) medium pH was 7.5 at 37°C. To get the low-pH medium, we added 1-M MES (Amresco, Solon (OH), USA) until pH = 6.5; to get the high-pH medium, we added 1-M Tris-base (Amresco) until pH = 8.5. Samples of culture supernatant were taken at 8–24-h intervals after inoculation and titrated for viral infectious activity in the VeroE6/TMPRSS2 cell line by the standard TCID50 method.

## Data Availability

The datasets presented in this study can be found in online repositories. The names of the repository/repositories and accession number(s) can be found below: https://gisaid.org/, KLU-RII-MH107906S.
