# Supplementary material for: A highly divergent sample from a nearly extinct SARS-CoV-2 lineage in a patient with long-term COVID-19
Source: Front Cell Infect Microbiol. 2025 Sep 19;15:1623390. doi: 10.3389/fcimb.2025.1623390 (PMC12491319; doi:10.3389/fcimb.2025.1623390)
Supplement: Supplementary file 1 [file Table1.pdf]

SUPPLEMENTAL TABLE

Data Availability

GISAID Identifier:	EPI_SET_250905tq
DOI:	https://doi.org/10.55876/gis8.250905tq

All genome sequences and associated metadata in this dataset are published in GISAID's EpiCoV database. To view the contributors of each individual sequence with details such as accession number, Virus name, Collection date, Originating Lab and Submitting Lab and the list of Authors, visit EPI_SET_250905tq

Data Snapshot

EPI_SET_250905tq is composed of 58,364 individual genome sequences.
The collection dates range from 2020-02-03 to 2023-03-13;
Data were collected in 165 countries and territories.
